# Variation in nest-building behaviour in birds: a multi-species approach

**DOI:** 10.1098/rstb.2022.0145

**Published:** 2023-08-28

**Authors:** Daniela M. Perez, Lilian T. Manica, Iliana Medina

**Affiliations:** ^1^ Max Planck Institute of Animal Behavior, Universitätsstraße 10, Konstanz, 78464, Germany; ^2^ Departamento de Zoologia, Universidade Federal do Paraná, Curitiba, Brazil; ^3^ School of BioSciences, University of Melbourne, Victoria 3056, Australia

**Keywords:** behavioural flexibility, innovative behaviour, environmental change, phylogenetic conserved traits, bird nest morphology

## Abstract

Researchers have long suggested that animals with greater behavioural flexibility will be more likely to survive in face of environmental changes. However, it is unknown how this varies across species. Nest building is a behaviour directly related to the reproduction and survival of species by conferring protection from external environmental conditions. The study of nests offers a window into the behaviour of birds, and variation in nest morphology is necessarily linked to variation in building behaviours. We test whether variation in nest morphology is phylogenetically conserved by using data on nest morphology from 55 passerine species (>700 specimens) and measuring intraspecific variability in nest structure. We found that species mean and within-species variation in nest morphology are phylogenetically conserved, and that species with domed nests presented higher levels of nest morphology variation than cup nest species. We also revealed that the capacity of species to present innovative behaviours is not linked with how they vary nest morphology. Moreover, we revealed that nests from species with larger variation in clutch size and that are built by single parents are more variable. Our results help in the understanding of how behaviour and extended phenotypes evolve, and highlight the importance of exploring the phylogenetic history of behavioural flexibility when trying to predict the capacity of species to respond to novel challenges.

This article is part of the theme issue ‘The evolutionary ecology of nests: a cross-taxon approach’.

## 1. Introduction

In the past decade it has been widely acknowledged that animal behaviour could be critical in future scenarios related to habitat loss, pollution, or climate change [1,2]. Behaviour can help species to cope with human-induced change, and responses can involve learning to avoid novel predators, seeking for novel places for shelter or shifting foraging patterns [3–5]. The capacity of animals to vary their behaviour in response to novel conditions (through genetic adaptation or plasticity) has been linked to different measures of ecological and evolutionary success. In birds, for instance, species that are capable of adapting can show higher invasion success and reduced extinction risk [6,7]. Recently, it was shown that bird species with larger brain sizes have been less affected by the damaging effects of climate change, and their adaptive responses (via changes in body size) have been minor relative to species with potentially less behavioural flexibility [8].

In birds, one of the least studied but potentially most important types of behaviours is nest building [9]. Most birds spend days or even weeks building structures that are fundamental in the protection of their progeny, providing the appropriate microclimate for development and shielding eggs and chicks from predators and parasites [10–12]. Nest building has been considered for many years an extremely conserved trait, with nest traits often being used to identify species and whole families [13]. Past studies on the macroevolution of nests have used general nest types at the family or genus level to represent entire clades [14,15]. The classification of nests into general nest types (open cups or domed nests built inside or outside cavities) facilitates analyses at a broad scale, but such classification is oversimplistic and we are now aware that there are different levels of variation in nest morphology. For instance, a recent analysis showed that many families present variation in nest types, and at least 60 species are known to build two different types of nests (e.g. with or without a roof) [16]. The view that nest building is an instinctive and inflexible behaviour has led to a systematic disregard of variation in nest architecture within species, but it is now acknowledged that information from the environment and experience can affect nest building decisions [17,18].

Birds may vary nest architecture (nest-building behaviours) according to ecological factors, which could be critical in future changing environmental scenarios. Nest size, lining and composition can vary depending on local habitats (reviewed in [19]). For instance, it was recently shown that smaller nests are built in warmer climates, suggesting that environmental variables can drive the evolution of nest size [20]. Within species, it has also been shown that blackbirds (*Turdus merula*) build larger nests and with thicker walls in cooler temperatures [21] and a similar pattern has been found in blue tits (*Cyanistes caeruleus*), great tits (*Parus major*) and other North American species [22,23]. Within individuals, southern masked weavers (*Ploceus velatus*) and village weavers (*Ploceus cucullatus*) build smaller nests through the breeding season, which could reflect experience and higher efficiency in nest building [24]. New helmeted honeyeaters (*Lichenostomus melanops*) also build smaller nests with thinner walls as the breeding season progresses, potentially because temperatures increase [25].

Flexibility in behaviours could be key to predicting resilience in future scenarios. There is initial evidence that species capable of innovating are at lower risk of extinction [7], and species with higher behavioural flexibility have been less negatively affected by the effects of global warming [8]. Although these examples point at individual flexibility in behaviour, quantifying intraspecific behavioural variation can also inform on how labile species are, and their potential to respond to environmental change [26–29]. Quantifying behavioural variation, however, is not a trivial task as extensive hours of field observations are usually needed. For nesting behaviours, a way to overcome this hindrance is by quantifying an organism's extended phenotype (nests), as these are ultimately a reflection of individual behaviours [9]. Thus, although extended phenotypes are not direct behavioural observations, they provide an archive of an organism's past behaviours. Birds' nests are one of the most exemplary extended phenotypes [9], and although studies have shown that nest traits such as size, volume or wall thickness are under selection and have important functions for thermoregulation, sexual selection or antipredation (e.g. [19,30,31]), we know very little about variation and flexibility in nest morphology within species. Measuring levels of trait variation across a range of species is a difficult task, but museums offer an incredible (and underused) resource. Nest collections hold thousands of records collected across years and geographical regions. Although there are drawbacks from using collected nests (e.g. degradation through time, uneven sampling across space and time), they offer our best chance to gain a first approximation of the levels of variation in nest morphology that are exhibited among a wide range of species.

In the present study, we access a comprehensive museum nest collection to explore the levels of intraspecific variation in nest morphology. We assess nest-building variation by measuring the dimensions of 4 traits of nest morphology, nest height, cup depth and internal and external cup diameter. We aim to understand: (1) if the variation in nest traits within species is restricted to a few species or widespread, (2) whether some nest traits (i.e. cup depth or internal cup diameter) are more variable than others, (3) if intraspecific variation in nest morphology is evolutionarily conserved. Moreover, given that nest building is a type of behaviour, we also explore whether species with high flexibility in nest morphology also present high flexibility in other types of behaviours. To do so, we test (4) whether variation in nest-building behaviour within species is linked to previously published scores of behavioural innovation; and (5) how intraspecific nest morphology variation is linked with intraspecific variation in clutch size and with who builds the structure—single or both parents. Answering such questions constitutes an important first step to evaluate the potential that nesting behaviours could have in a changing world, and the capacity of species to reproduce in modified environments.

## 2. Methods

### (a) Data collection

**Figure 1.**
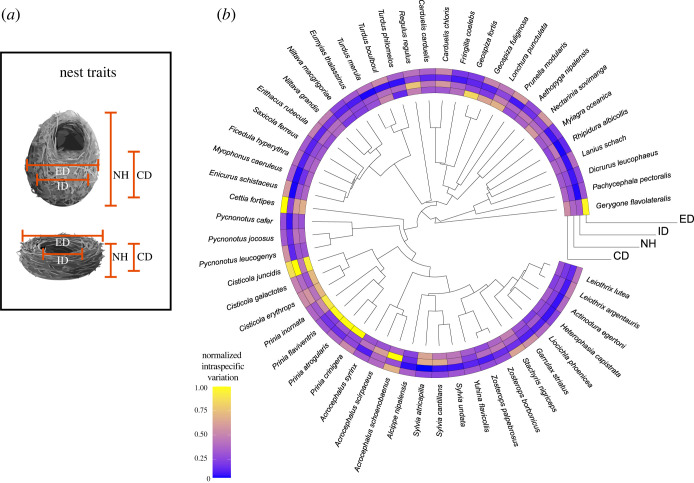
Illustrations for (*a*) the measured nest traits, cup depth (CD), nest height (NH), internal (ID) and external cup diameters (ED), on dome nest and open cup nests; (*b*) nest morphology intraspecific variation for the four nest traits, plotted in the phylogenetic tree. The species' intraspecific variation values represented are standardized by the maximum value for each nest trait. Intraspecific variation values range from yellow (highest building variation) to blue (lowest building variation).

To quantify intraspecific variation in nest morphology, we assessed one of the largest ornithological nest collections in the world, the Natural History Museum in Tring, UK [32] from February to March, 2020. We measured 745 nests from 55 species of 20 Passeriformes families (8 to 25 nests per species, average = 13.54 ± 4.5). A single researcher measured nest height, cup depth and internal and external cup diameter (three times for each nest) by using a divider and a dial caliper (figure 1*a*). For species presenting asymmetrical nests, measurements of the largest and shortest lengths were included and that did not affect repeatability scores (<0.9; electronic supplementary material, table S1). The traits measured in this study match the ones collected by Vanadzina *et al.* [20] with the exception of nest wall thickness. Like in the present study, the authors harvested data from the same museum collection, although in our data collection species were chosen based on the number of nests available (minimum eight nests per species). Good nest condition (e.g. no flattened specimens or loose material) and the availability of information regarding the year and location of their collection were also essential. Nests were open cups or domes derived mostly from species that build nests on vegetation and not in cavities (from the total of 55 species included in the study, 12 species nest in cavities; electronic supplementary material, file S2).

To quantify how variable nests are within each of the 55 species and between traits, we log-transformed all nest measurements and calculated the variation coefficient (standard deviation/mean) for each nest trait. Unlike the standard deviation of nest traits that are tied to their means (larger nests have larger variation), the variation coefficient is an estimator that can be used to control for the effect of specimen absolute sizes (i.e. [33,34]) as larger values indicate higher variation in specimens around the species mean (higher intraspecific variation).

### (b) Analyses

#### (i) Phylogenetic signal

To reveal to what extent intraspecific variation in nest building is correlated with phylogenetic history, we obtained the phylogenies from https://birdtree.org/ using the Hackett backbone [35] and calculated the phylogenetic signal *λ* [36] for each of the four nest measures using the packages Phytools [37] and Picante [38] in the R 3.6.3. environment [39]. The phylogenetic signal is a measure of the tendency of traits between more closely related species to resemble each other, by assuming a stochastic Brownian motion of trait evolution proportional to branch lengths on the phylogenetic tree [40]. Under phylogenetic signal the estimated parameter *λ* approaches 1, and under other modes of evolution that do not follow the phylogeny, the value of *λ* is lower. For each of the four nest traits, we separately tested the significance of the estimate of *λ* of the maximum clade credibility (*λ*_MCC_) phylogenetic tree using likelihood ratio tests and generated the 95% High Density Probability (HDP) interval calculated from the set of 1000 phylogenies. In addition to quantifying the phylogenetic signal in the intraspecific variation in nest morphology, we also measured the phylogenetic signal of both the mean nest traits (log-transformed) and absolute body size, as an informative point of comparison. It is relevant to separately analyse intraspecific variation and species means to understand how these values are associated (or not) and how labile mean nest traits are at the interspecific scale.

#### (ii) Accounting for sampling variation

Given that samples were collected across different numbers of years and locations, we first tested whether these two factors were linked to higher variation in nest morphology within species (intraspecific variation value above). We ran phylogenetic generalized least-squares regressions (PGLS) for each nest trait [41,42] using the pgls function (Caper package; [43]). These were run for the MCC phylogenetic tree and the 1000 phylogenies to generate the HDP interval and account for phylogenetic uncertainty. For each species, the within-species variation in each nest trait was the response variable, and the range of years and latitudes of the sampled nests were used as fixed explanatory variables.

#### (iii) Predictors of variation in nest morphology

We aimed to reveal whether intraspecific variation in nest building—as measured by the intraspecific variation values above—is related to measures of behavioural flexibility, average species body size, the type of nest built by a species (open cup or domed), the species clutch size range and whether both or single parents build the nest. Nest type is an essential component of such models as it has high potential to affect nest traits, such as dome nests having deeper cups. A general description of species body mass, nest type, clutch size range and who builds the nest were obtained from the online ornithological platform Birds of the World (https://birdsoftheworld.org/bow/home; [44]), while the measure of behavioural flexibility was obtained from the binary coefficient developed by Ducatez *et al*. [7], who collated information from an extensive database of field behavioural observations and classified species that have been reported presenting innovative foraging behaviours or not. This index of innovation is associated with lower extinction risk and stable or increasing population trends [7]. We ran a series of PGLS models for each nest trait and across 1000 phylogenies to generate the HDP interval and account for phylogenetic uncertainty. Within-species variation in nest morphology was pitted as the response variable, and innovation coefficient (0 or 1), body mass and nest type (open cup or dome) as fixed explanatory variables (sample on these predictors, *n* = 38). To test the link between variation in each nest trait, whether both or single parents build the nest and clutch size range for each species, we performed a separate analysis in a subset of data due to lack of model convergence from the lower sample size (sample whether both or single parents build the nest, *n* = 33).

## 3. Results

Our sampling comprised species with a wide range of body mass (from 6 to 183 g) and nest sizes. Mean nest traits ranged among species from a cup depth of 2.5 cm (*Prinia flaviventris*) to 9.2 cm (*Lonchura punctuata*), nest height of 3.7 cm (*Zosterops palpebrosus*) to 20.8 cm (*L. punctuata*), internal cup diameter of 2 cm (*Gerygone flavolateralis*) to 10.4 cm (*Myophonus caeruleus*) and external cup diameter of 5.2 cm (*Cisticola jucidis*) to 16.9 cm (*Turdus philomelos*). Nest intraspecific variation ranged from 2% (*Turdus philomelos*) to 15% (*Prinia crinigera*) for cup depth, 2% (*Alcippe nipalensis*) to 14% (*Acrocephalus schoenobaenus*) for nest height, 1% (*Sylvia atricapilla*) to 27% (*Cisticola jucidis*) for internal cup diameter and 1% (*Riphidura albicollis*) to 9% (*Cethia fortipes*) for external cup diameter (figure 1*b*). The internal cup diameter had the highest range of building variation between species, reaching almost twice the variation as the slightly correlated structures external cup diameter and cup depth (electronic supplementary material, figure S1). All traits presented similar average levels of intraspecific variation, with cup depth being the highest (mean intraspecific variation value: 6.9% ± 3% for cup depth, 5.3% ± 2% for nest height, 4.6% ± 4% for internal cup, 3.7% ± 1% for external cup). Species with high variation in one trait do not necessarily present high variation in other traits (maximum *r*^2^ = 0.53, between variation in cup depth and variation in internal cup diameter; electronic supplementary material, figure S1) and were subsequently analysed separately.

### (a) Phylogenetic signal

**Figure 2.**
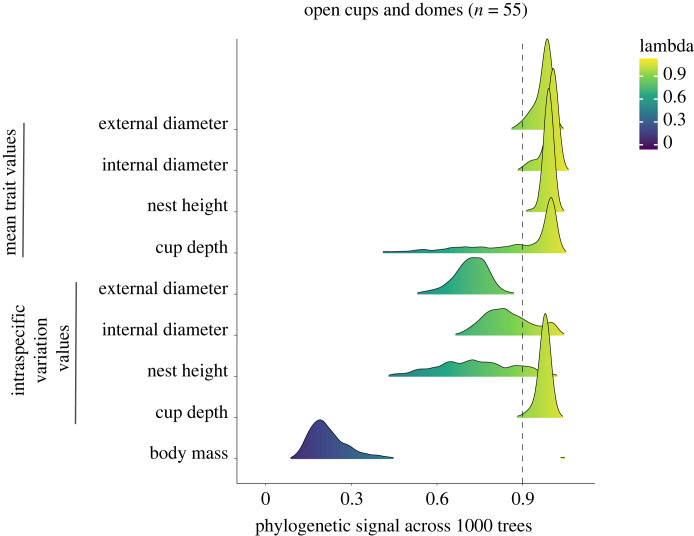
Forest plot indicating the distribution of values of phylogenetic signal across 1000 trees. Each distribution curve represents the distribution for a distinct nest trait and body mass. The distributions of phylogenetic signals for the nest traits are separately plotted for mean nest trait values and intraspecific variation values. Lambda is indicated on the *x*-axis and cut-off value of 0.9, representing high phylogenetic signal, is indicated by the dashed line. The colour range indicates lambda values from yellow *λ* = 1 (high) to blue *λ* = 0 (low). Complete data with open cups and dome species (*n* = 55).

**Figure 3.**
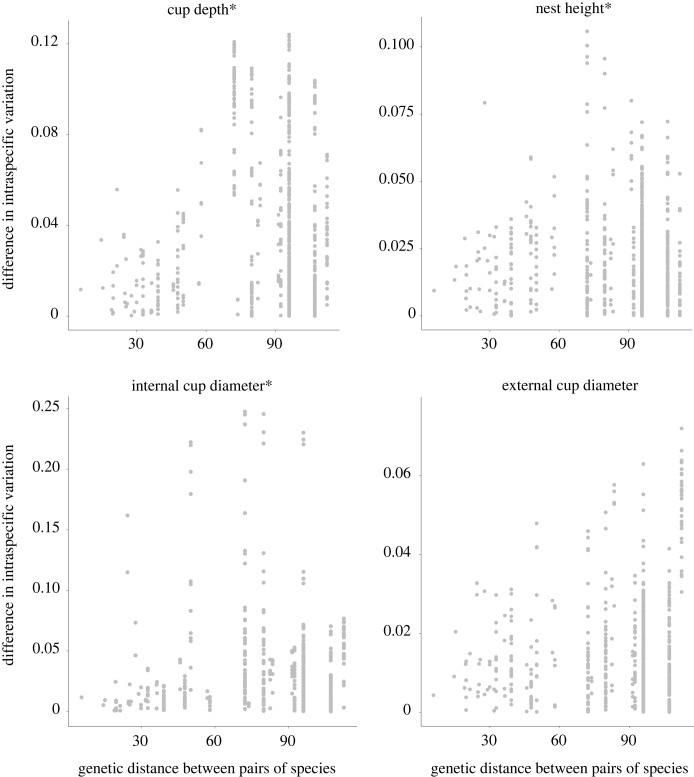
Difference in intraspecific nest-building variation in relation to the genetic distance (millions of years) between pairs of species for each of the four nest traits studied (cup depth, nest height, internal and external cup diameters). Symbol * indicates the nest traits that presented significant phylogenetic signal, *p* < 0.05.

Intraspecific variation in cup depth, nest height and internal cup construction is phylogenetically conserved (have high phylogenetic signal – *λ* values close to 1) across species (*λ*_mcc_ = 0.97 [HDP 0.92–1], *p*_mcc_ < 0.001; *λ*_mcc_ = 0.92 [HDP 0.5–1], *p*_mcc_ < 0.001; *λ*_mcc_ = 0.99 [HDP 0.71–1], *p*_mcc_ < 0.001, respectively; figures 2 and 3). Although the intraspecific variation in external cup diameter showed a relatively high phylogenetic signal, it was not significantly different from zero, possibly following an evolutionary path independent from the one indicated in the phylogeny (*λ*_mcc_ = 0.78 [HDP 0.6–0.83], *p*_mcc_ = 0.06; figures 2 and 3). Mean nest traits also exhibited extremely high values of phylogenetic signal (all *λ* > 0.99; electronic supplementary material, table S2). Species body mass showed a much lower phylogenetic signal than any mean nest trait or any measure of intraspecific variation in nest traits (*λ*_mcc_ = 0.38, [HDP 0.12–0.39] *p*_mcc_ = 0.15, figure 2; and also, for tarsus length *λ*_mcc_ = 0.41, *p*_mcc_ = 0.14). We also separately analysed only open cups not in cavities (51% of the data) and found that a high phylogenetic signal in intraspecific variation and mean nest measurement is maintained for all traits (electronic supplementary material, figure S2 and table S2). However, these results were not statistically significant except for cup depth mean (*p* = 0.04). The phylogenetic signal for external cup diameter shifts considerably from the dataset with all types of nests to the dataset of open cups not in cavities: from 0.99 to < 0.001 in mean values (*p* = < 0.001 and 1) and from 0.78 to 1 in intraspecific variation values (*p* = 0.06 and < 0.01; figure 2, electronic supplementary material, figure S2 and table S2).

### (b) Predictors of variation in nest morphology

Intraspecific variation in nest traits was not related to latitude or year span except for one trait, where variation in internal cup diameter tended to be slightly higher when collected across larger latitude spans, although not significant (*β*_mcc_ = 0.009 [HDP 0.009–0.013], *t*_mcc_ = 1.97 [HDP 1.88–2.74]; *p*_mcc_ = 0.053 [HDP 0.005–0.056], electronic supplementary material, table S3). Given that the effect of collection location and year on variation was negligible in our sample, we used the values of intraspecific variation as response variables for all nest traits in the posterior PGLS analyses. Species capacity to present innovative behaviours (*λ*_mcc_ = 0.66 [HDP 0.8–0.2], *p*_mcc_ < 0.01) is not correlated to intraspecific nest building variation in any of the traits (table 1). Species body mass was also not linked to intraspecific nest variation in any of the traits, while species with dome nests present higher intraspecific variation in cup depth than species with open cup nests (cup depth: *β*_mcc_ = 0.053 [HPD 0.049–0.057], *p*_mcc_ < 0.001 [HPD < 0.0001–0.0001], *t* = 4.765 [HPD 4.2–5.5], table 1). Lastly, species with larger variation in clutch size present more variable nest height, internal and external cup diameters (nest height: *β*_mcc_ = 0.008 [HPD 0.008–0.009], *p*_mcc_ = 0.002 [HPD 0.001, 0.002], *t* = 3.359 [HPD 3.348, 3.747]; internal cup diameter: *β*_mcc_ = 0.009 [HPD 0.008, 0.011], *p*_mcc_ = 0.034 [HPD 0.014, 0.059], *t* = 2.221 [HPD 1.956, 2.580]; external cup diameter: *β*_mcc_ = 0.005 [HPD 0.005, 0.006], *p*_mcc_ < 0.001 [HPD < 0.001, 0.001], *t* = 4.416 [HPD 3.632, 5.834], table 2). We also found that when single parents are involved in nest building they show higher intraspecific variation in internal and external cup diameter than species in which both sexes contribute in building (internal cup diameter: *β*_mcc_ = 0.032 [HPD 0.031, 0.039], *p*_mcc_ = 0.026 [HPD 0.010, 0.041], *t* = 2.342 [HDP 2.124, 2.708]; external cup diameter: *β*_mcc_ = 0.008 [HPD 0.009, 0.012], *p*_mcc_ = 0.032 [HPD 0.003, 0.04], *t* = 2.248 [HDP 2.038, 3.059], table 2).

**Table 1.** Results for the separate PGLS models for each nest trait intraspecific variation coefficient examining the relationship between innovation index, species body mass and nest type. Main values were generated from models using the MCC phylogenetic tree, and HDP intervals generated from a set of models across 1000 phylogenetic trees are represented in brackets. Intercept entails species that do not present innovative behaviours and cup nests. Results of all trees are presented in the supplementary file S3.

nest trait	estimate intercept	*t*	*p*	estimate innovation	*t*	*p*	estimate body mass	*t*	*p*	estimate nest type	*t*	*p*
cup depth	0.051	4.213	< 0.001^a^	0.003 (0.001, 0.009)	0.299 (0.062, 0.92)	0.767 (0.31, 0.87)	< 0.001 (< −0.0001, 0.0001)	0.287 (< −0.001, 0.46)	0.776 (0.65, 0.99)	0.053 (0.049, 0.057)	4.765 (4.2, 5.5)	< 0.001^a^ (<0.0001, 0.0001)
nest height	0.057	3.926	< 0.001^a^	< 0.001 (−0.003, 0.003)	0.027 (−0.44, 0.36)	0.978 (0.66, 0.99)	< −0.001 (< −0.0002, < −0.0001)	−0.411 (−1.19, −0.21)	0.684 (0.18, 0.73)	−0.007 (−0.012, −0.006)	−0.581 (−1.14, −0.52)	0.565 (0.26, 0.60)
internal cup diameter	0.044	1.661	0.106	−0.008 (−0.021, −0.006)	−0.541 (−1.39, −0.402)	0.592 (0.172, 0.69)	< −0.001 (−0.0003, −0.0001)	−0.679 (−0.78, −0.51)	0.501 (0.44, 0.61)	0.025 (0.025, 0.043)	1.128 (1.08, 2.33)	0.267 (0.002, 0.23)
external cup diameter	0.032	8.509	< 0.001^a^	−0.0052 (−0.0053, −0.0052)	−1.129 (−1.130, −1.129)	0.267 (0.266, 0.267)	< 0.001 (< −0.0001, < −0.0001)	1.159 (1.159, 1.160)	0.254 (0.254, 0.255)	0.009 (0.009, 0.01)	1.993 (1.992, 1.993)	0.054 (0.054, 0.055)

^a^Values for *p* with test significance equal to or below 0.05.

**Table 2.** Results for the separate PGLS models for each nest trait intraspecific variation coefficient examining the relationship between range of clutch sizes and building effort (single or both parents). Main values were generated from models using the MCC phylogenetic tree, and HDP intervals generated from a set of models across 1000 phylogenetic trees are represented in brackets. Intercept entails species in which both parents contribute to nest building. Results of all trees are presented in the supplementary file S3.

nest trait	estimate intercept	*t*	*p*	estimate clutch size range	*t*	*p*	estimate building effort	*t*	*p*
cup depth	0.056	3.647	<0.001^a^	0.003 (0.002, 0.005)	1.318 (1.027, 1.906)	0.199 (0.052, 0.291)	−0.008 (−0.004, 0.004)	1.064 (−0.449, 0.537)	0.296 (0.602, 0.999)
nest height	0.029	2.475	0.019^a^	0.008 (0.008, 0.009)	3.359 (3.348, 3.747)	0.002^a^ (0.001, 0.002)	0.008 (0.007, 0.013)	0.915 (0.877, 1.793)	0.367 (0.083, 0.387)
internal cup diameter	< −0.001^a^	< −0.001	0.999	0.009 (0.008, 0.011)	2.221 (1.956, 2.580)	0.034^a^ (0.014, 0.059)	0.032 (0.031, 0.039)	2.342 (2.124, 2.708)	0.026^a^ (0.010, 0.041)
external cup diameter	0.015	2.192	0.036^a^	0.005 (0.005, 0.006)	4.416 (3.632, 5.834)	< 0.001^a^ (< 0.001, 0.001)	0.008 (0.009, 0.012)	2.248 (2.038, 3.059)	0.032^a^ (0.003, 0.04)

^a^Values for *t* with test significance equal to or below 0.05.

## 4. Discussion

In the present study, we use museum collections to explore and quantify levels of intraspecific variation in nest morphology across 55 species. Overall, we found that there is considerable variation across species in their levels of intraspecific variability in all nest traits. Some species build extremely uniform nests, with very low variation levels (< 2% across specimens), while others exhibit high levels of variation (> 20%). Phylogenetic history explains these patterns to a considerable degree, where closely related species exhibit not only similar mean trait values but also similar levels of variation in all nest traits. We found no association between species body mass and variation in nest morphology. Nests built by species with higher levels of behavioural innovation were also no more variable in any nest trait. However, cup depth was more variable in species building dome nests, species with more variable clutch sizes built nests in which heights and internal and external cup diameters were more variable, and single parents tended to build nests with more variable internal and external cup diameters.

Contrary to our expectations, species with nests collected across a wider range of years and locations were not more variable, and sampling did not explain intraspecific variation. First, this is an indication that the nest collection is well preserved and variation is not due to material loss through the years. Second, this also suggests that variation in the morphology of our sampled nests is not necessarily driven by local adaptation, as nest morphology should be more variable if nests had been collected from a wider variety of environments. Nevertheless, several studies have previously pointed at associations between nest morphology (thermal properties) and environmental factors, particularly local temperature (i.e. [20,31,45]). For example, a study in Canada found that several species had wider internal nest-cup diameters in colder environments [22]. Experimental studies have also shown direct effects between temperature and nest size. For example, zebra finches build larger nests under lower temperatures, which confers higher reproductive success [18]. An explanation for our findings is that some species are able to present adaptive changes across their distributions, but this is not a widespread response across most species. It is also possible that latitudinal ranges may be too large to detect finer environmental gradients or traits may be genetically constrained in some species. This may be the case for the European robin, *Erithacus rubecula,* with generally low nest-building variation (4 to 5%) in nests sampled from distant latitudes (latitudinal range of 24°, from 28.3° to 52.3°), agreeing with the results of a previous study by Cerezo and Deeming [46] which found that insulation and dimensions do not change across latitudes.

We acknowledge that there may be other unexplored external variables that can lead to individual variation in nest construction. For example, body mass could potentially correlate to variation in nest morphology. However, we found no such association, although information on within-species variation in body mass would be more relevant for this investigation. It is also possible that the variation captured by our metric is a by-product of individual nest-building styles driven by variation in personality, individual quality, cognition or experience and hence not necessarily linked to local conditions [9,47]. For example, nest morphology can change throughout a breeding season and nests may be used primarily for courtship; this is the case of *Cisticola jucidis*, which also showed the highest overall nest variation in the present study [48]. Moreover, birds may associate nest morphology to their reproductive success, such as preferences in the zebra finch (*Taeniopygia guttata*) for nest material colour that is linked to past successful broods [47]. Nesting preferences, although not necessarily morphological *per se* (i.e. nest site), may also be nongenetic when transmitted through social learning both intra- and interspecifically, and lead to variation among individuals [49,50]. For example, nesting site preferences in the migrant species *Ficedula* sp. can change after observing nesting behaviour of the ecologically similar and resident *Parus* sp. [50].

The examples above and many others comprise a large body of evidence that intraspecific variation in nest morphology can have intricate links with local adaptation or plasticity. Yet, the high values of phylogenetic signal found in the present study also show that evolutionary history plays an important role in explaining flexibility in nest morphology. We found that not only are the overall means of nest structures phylogenetically conserved, but also how much they vary from that value, meaning that both the pattern and the building flexibility are characteristic to a species. Moreover, the phylogenetic signal estimated for intraspecific variation was considerably higher than the phylogenetic signal of morphological traits such as body mass (figure 2). Behavioural traits are often considered to be more labile than morphological traits [40,51,52], but our results show that nest building might be an exception, and nest traits (and their variation) can be highly conserved across species, independent of nest type. It is possible that closely related species exhibit similar traits and levels of flexibility because nest-building has a strong neural underpinning [53], constraining species’ capacities to alter nest construction. Thus, although there are caveats with our quantification of intraspecific variation given the potential noise in our dataset (i.e. no control over sampling design), the high levels of phylogenetic signal both in the mean trait values and in the intraspecific variation indicate that these estimates are probably quite conservative, and could be higher.

In pursuit of a better understanding of the ecological factors driving nest morphology variation evolution, we tested the links between some important ecological aspects and nest morphology. However, besides phylogeny, we generally found weak predictors. First, measures of species behavioural innovation were not higher in species with higher intraspecific variation in nest morphology. The phylogenetic signal of the innovation index was only slightly lower than the phylogenetic signals for the intraspecific variation in all nest traits, suggesting that innovation and nest morphology are mainly driven by phylogeny and there is no evidence that lineages with higher innovation capacities have evolved higher nest morphology variation. Thus, it is possible that species' ability to respond to novel environmental changes is not reflected in higher plasticity in nest construction, although the innovation coefficient used here is based on foraging behaviour observations and using a more related measure of innovation to nest building variation could show a positive association. Second, we detected higher intraspecific variation in cup depth in species that build dome nests as opposed to species that build open cup nests. It is intuitive that domes have higher structural variation because they are usually suspended from branches and grasses, although we would expect the same effect on nest height, which we did not observe. It is known that nest base is positively related to clutch size in many bird species, but this trend is regardless of nest type [20,54]. Varying cup depth could be due to alterations in nest lining, and the explanation for the greater cup depth variation in dome nests than in open cup nests could be that the walls of dome nests simply allow for such variation without posing a risk to nestlings from falling. Our results also showed that nests from species with higher variation of clutch sizes overall build more variable nests. This result is expected since nests should vary to accommodate the eggs laid and also to withstand nestling activity. We suggest that parents optimize nest building effort according to clutch size (smaller nests when laying fewer eggs). Lastly, we found that nests built by a single parent (male or female) have more variable internal and external cup diameters than nests built by couples. This was unexpected, since a previous study comparing several species [31] reported that nests are on average larger when built by a couple as opposed to a single parent (female), meaning there could be more opportunity for variation in nests built by couples. We suggest that if male and female do not collaborate and follow the same blueprint of nest construction (which may be determined by genes or communication), they would not be able to build a nest together, leading to low variation in nest architecture. Moreover, nest building is not only influenced by natural selection: both monogamous and non-monogamous bird species can use nests to signal mate quality and nest morphology variation can be under the pressures of sexual selection, although this case would mostly apply when nests are built by males [31]. Thus, it is possible that competition and/or honest signalling by single parents result in higher behavioural variation in nest construction and morphology [55].

In conclusion, bird nests have been the subject of extensive research for many years, but broad-scale studies have solely focused on species-level values, ignoring intraspecific variation [14–16], despite this being the raw material of natural selection and adaptation [56,57]. Our study shows that information from museum collections represents a valuable opportunity to gain understanding on levels of variation in nest building across a wide range of species. It also shows that phylogenetic history should be taken into account when determining the ability of species to respond flexibly to novel challenges imposed by anthropogenic impacts. Future studies addressing trait variation to understand the extent to which nest building relates to other selective pressures, such as adaptations to anthropogenic change (i.e. urban environments), will help better grasp how much of this behaviour is regulated by species ecology and external influencers.

## Data Availability

The computing codes and datasets supporting this article have been uploaded as part of the electronic supplementary material [58].
